# Legitimizing user knowledge in mental health services: Epistemic (in)justice and barriers to knowledge integration

**DOI:** 10.3389/fpsyt.2022.981238

**Published:** 2022-08-25

**Authors:** Katarina Grim, Hilda Näslund, Conny Allaskog, Jessica Andersson, Elisabeth Argentzell, Kjell Broström, Filippa Gagnér Jenneteg, Mårten Jansson, Ulla-Karin Schön, Petra Svedberg, Sara Svensson, Sonny Wåhlstedt, David Rosenberg

**Affiliations:** ^1^Department of Social and Psychological Studies, Karlstad University, Karlstad, Sweden; ^2^Department of Social Work, Umeå University, Umeå, Sweden; ^3^NSPH National Association, Stockholm, Sweden; ^4^NSPH Skåne, Malmö, Sweden; ^5^Department of Health Sciences, Lund University, Lund, Sweden; ^6^NSPH Stockholm, Stockholm, Sweden; ^7^NSPH Västra Götaland and Gothenburg, Gothenburg, Sweden; ^8^Department of Social Work, Stockholm University, Stockholm, Sweden; ^9^School of Health and Welfare, Halmstad University, Halmstad, Sweden

**Keywords:** mental health services, user involvement, co-production in research, epistemic injustice, user organizations, implementation, recovery

## Abstract

Including the voices and knowledge of service users is essential for developing recovery-oriented and evidence-based mental health services. Recent studies have however, suggested that challenges remain to the legitimization of user knowledge in practice. To further explore such challenges, a co-production study was conducted by a team of researchers and representatives from user organizations in Sweden. The aim of the study was to explore the barriers and facilitators to the legitimacy of user knowledge, as a central factor in sustainably implementing user influence in mental health practice. A series of workshops, with representatives of mental health services and user organizations were conducted by the research team to explore these issues. The analysis built on the theoretical framework of epistemic injustice, and the underlying aspects, testimonial, hermeneutic and participation-based injustice, were utilized as a framework for a deductive analysis. Results suggest that this is a useful model for exploring the complex dynamics related to the legitimacy of user knowledge in mental health systems. The analysis suggests that the legitimacy of user knowledge is related to the representativeness of the knowledge base, the systematic formulation of this knowledge in applicable methods, access to resources and positions within the mental health system and participation in the process of integrating this knowledge-base in mental health contexts. Legitimizing user knowledge in practice additionally challenges mental health systems to support readiness for change in working environments and to address the power and role issues that these changes involve.

## Introduction

The inclusion of users' knowledge is recognized as an essential component of the delivery and quality development of health care and social support, both in a Swedish and international context (1, 2). In the mental health field, including the voices and knowledge of users is essential for developing recovery-oriented and evidence-based services. The value of user knowledge and user choice has been reinforced in the growing body of research on recovery that increasingly guides mental health systems internationally (3–5). The importance of integrating user involvement in the mental health service system is further underscored in national policy and guidelines.

Recent studies have, however, suggested that while there is a positive discourse regarding user involvement, challenges remain to user knowledge being legitimized in practice (6). Users' participation tends to be reduced to tokenistic levels, where users are disregarded as epistemic partners in collaborative knowledge processes (6–8). Commonly, users highlight issues of dependency on professionals and not being considered as capable and trustworthy collaborators in shared deliberation (6, 9, 10). While imbalances of knowledge validation and power are recognized as barriers to participation in many domains of care and support, several studies have reported that greater levels of disempowerment, stigma and coercion in mental health settings may amplify barriers to user participation (11, 12).

While user knowledge perspectives are widely recognized as valuable in service development and provision, it is still unclear *how* user knowledge is to be incorporated into welfare systems (13). Accordingly, drawing on the framework of epistemic injustice, the aim of the study was to explore the barriers and facilitators to the legitimacy of user knowledge, as a central factor in sustainably implementing user influence in mental health practice.

### User knowledge and the swedish mental health system

In Sweden, there are two primary actors in the mental health system. Social psychiatric services, provided by the municipalities, support people with mental health problems with residential support, occupational- and social activities, rehabilitation and case management. Psychiatric services, provided by the regional health care system, include inpatient treatment, psychotherapy, medication and outpatient care. Because of this division of responsibility, there is a continuing challenge in Sweden to coordinate these two service providers (14). Findings from previous studies indicate that user representatives provide a more holistic understanding of users' needs that contribute to developing structures for bridging gaps and methods for coordinating services (15).

The user movement in Sweden consists of a multiplicity of user-led organizations, connected to the domestic tradition of popular mass movements and supported in part with government funding (16, 17). The National Partnership for Mental Health (NSPH), an umbrella organization consisting of the country's largest service user associations in the field of mental health, has developed a number of initiatives that focus on systematically integrating the knowledge of users in services at individual, organizational and systemic levels. The development and implementation of User-Focused Monitoring (UFM), Peer Support workers in services, and tools for supporting personal recovery in the form of written materials or apps, represent practices developed to strengthen user influence and support the integration of user knowledge in practice.

### User knowledge and epistemic injustice

The Recovery framework underscores the holistic nature of mental illness, promoting more emphasis on the situated, experiential knowledge of service users (3, 4). Experiential knowledge has been portrayed as complex, layered and holistic (5, 18). It entails social, emotional and embodied experiences of living with and managing an illness, as well as experiences of stigma and vulnerability. The knowledge perspective of users is not merely based on personal experiences but is constructed through a collective process, which involves sharing and distilling various perspectives. This knowledge form is therefore both personal and collective in nature (19). Technological developments have contributed to information now being more readily available, providing people with access to research studies, medical guidance and public discussion forums (18). In accordance with these descriptions, we conceptualize user knowledge as not limited to knowledge acquired through personal experiences but as situated knowledge perspectives that are continually co-constructed through merging lived experiences with collectively shared knowledge and scientific (e.g., medical) knowledge.

Despite the focus on acknowledging users as bearers of valuable knowledge, there is no consensus however on what aspects of user knowledge should be considered legitimate knowledge. Typically, user knowledge continues to be considered anecdotal and hierarchies continues to place constraints on the inclusion of users' knowledge perspectives in welfare services. Recent literature, drawing attention to the epistemically complex aspects involved in integrating user knowledge in the context of mental health care, suggest that challenges can be brought to light by applying Fricker's (20) conception of epistemic injustice (6, 9, 10). The concept of epistemic injustice refers to an injustice done to people in their capacity as knowledge bearers, reasoners and questioners, in which their ability to take part in epistemic practices, such as providing knowledge to others (testifying) or making sense of their experiences (interpreting), is weakened (20).

As the description suggests, Fricker articulates two such wrongs: *testimonial injustice* and *hermeneutical injustice*. Testimonial injustice occurs when a persons' capacity as a reliable informant and conveyer of information and knowledge is breached. This devalued credibility is often due to identity prejudice. The speaker's membership in a negatively stereotyped group causes the hearer to view their accounts and arguments as less competent and sincere–and thus less trustworthy. Hermeneutical injustice occurs when there is a breach in shared conceptual, interpretative resources that puts people at a disadvantage when trying to make sense of their experiences. When shared modes of interpretation (such as concepts, ideas and narratives) are unavailable, these people are deprived of the capacity to use and develop the shared descriptive labels necessary for a mutual understanding of the phenomena they experience. Many theorists have expanded on the theory of epistemic injustice (21). One such elaboration, is the concept of *participant-based injustice* (22). Participant based injustice involves a (partial) exclusion of individuals or groups as collaborators in knowledge processes, i.e., in knowledge gathering, shared inquiry and deliberation, problem-solving and decision making.

Prior studies have illustrated that epistemic injustice is a valuable concept for analyzing barriers to the inclusion of user knowledge at an individual level (6, 9, 10). In this study, we apply these concepts to explore barriers to user knowledge integration in service development and provision.

## Methods

The study builds on a co-production design that included six researchers from various disciplines and seven user organization representatives, as members of a research team. The user movement representatives hold central positions within the NSPH and have wide-ranging experiences of user involvement initiatives. An ambition of the study has been to integrate co-production throughout the research process, moving beyond consultation and toward knowledge production in partnership (23). The goal was to create a collaboration that was based on our complementary expertise [cf. Fleming et al., (24), p. 711]. The members of the research team have therefore been involved in all stages of the study, from initial formulation of the research proposal, to the study design, data collection, analytical procedures and in the communication of research results, contributing with their own competence and perspective.

### Co-produced workshops

Utilizing a co-production design, the team developed an interview framework and conducted a series of digital workshops (due to the pandemic) with I) user representatives and II) mental health program directors and practitioners. In total, we carried out three workshops (each of which approximately 3 h long). Two of these targeted program directors and practitioners representing the mental health service system, and one targeted representatives of the user movement. In total, there were 28 participants in the workshops. (see Tables 1, 2).

**Table 1:** Representatives of the mental health service system–workshop I and III (*N* = 14).

Occupational	Quality development program director	6
	Department manager	3
	Unit manager	2
	Politician	1
	User influence coordinator	2
Region in Sweden	West	5
	East	3
	South	3
	Southeast	3

**Table 2:** User movement representatives–workshop II (*N* = 14).

Organization	NSPH (umbrella organization)	2
	Local NSPH associations	8
	Other local user organization	1
	User led enterprise	1
	Adult educational association	1
	User influence coordinator	1
Region in Sweden	West	4
	East	7
	South	1
	National	2

Participants were recruited through the network of the research team to form a purposive sample. The aim was to include individuals from a variety of regions in Sweden with substantial knowledge of the implementation of methods based on user knowledge in mental health practice. Most participants had experience of systematic user involvement attempts. In particular, the methods of UFM, The Recovery Guide and Peer support were discussed in the workshops.

- *The Recovery Guide* is a tool developed by the NSPH to support personal recovery. It is available as a printed format, mobile app and study circle. It is a workbook and the material builds on experiential knowledge of recovering from serious mental illness and presents recovery principles and strategies that can serve as tools for people attempting to participate in their care planning (25).
- *Peer support* involves people in recovery from mental illness who are trained and employed to offer support to others using psychiatric services due to mental health problems. In Sweden, the NSPH plays a central role in both the education, supervision and coordination of peer support (26).
- *UFM* is a method of reviewing care and supports, performed by people with experiential knowledge of mental ill health (27, 28). In Sweden, user organizations often organize UFM and train user monitors in evaluation methods. Based on a commission from a service organization, teams of user monitors conduct an evaluation of a service site or intervention from a user perspective (28, 29).

The user movement representatives and researchers in the team, as well as the interviewees participating in the workshops, are not a representative sample for all who we might have spoken with and there are certainly additional viewpoints that should be attended to. Smaller regions and cities or towns may, for example, not even have an organized user movement. While not representative of all perspectives, the participants were chosen based on their experience of these methods or other formalized, knowledge delivery projects involving user knowledge and influence.

The representatives from the mental health service system consisted of those representing municipal social psychiatric services and those representing regional psychiatric services. They were either higher-level department managers, or responsible for specific services, still others had a broad responsibility for quality development of services, including in these cases, a focus on user influence.

The user movement representatives included individuals who all had a specific role in the development and implementation of user influence initiatives. They were typically board members of either specific disability groups or the national association.

The interview framework was developed in the research team where we had introduced and discussed the theoretical framework related to epistemic injustice, and the study's ambition to focus on the “knowledge-question” and not simply implementation strategies. The resulting interview guide focused on exploring aspects of user knowledge in relation to the aim of user involvement, the implementation of different user involvement strategies, the effects of methods on the legitimacy of user knowledge and future ambitions.

Each workshop started with a joint introduction and discussion with all participants. In a next step, participants were divided into groups of 4–5 individuals, formed (by first, second and last author) to include a variety of perspectives representative of the total sample. Accordingly, heterogeneity was sought with respect to geographic location as well as forms of and roles in organizations. To conclude, a joint discussion was conducted, where participants shared and reflected on the issues discussed in the smaller group. During the workshops targeting officials and practitioners, the researchers and the user movement representatives of the research team were teamed up to share the interviewer role. The workshops directed primarily toward representatives of the user movement were also co-led but more directed by the professional researchers since the dual role of the research team members had to be acknowledged (30).

The workshops were recorded and transcribed verbatim. Regarding ethical considerations, verbal informed content was obtained from all workshop participants and no information trackable to unique individuals has been included in our results. Since no sensitive data was collected, ethical approval was not required.

### Co-produced analysis

The recordings were watched and analyzed in mixed researcher/user representative pairs using a live-coding (31) consensus model (32). This analytical approach means that the workshops were coded while watching the film, a method that may support the preservation of the participant voice in group interviews (31). This was considered a fitting approach for our co-production design, where some of the participants are not trained in research methods. It further contributed to a dialogue that served to involve the complementary expertise of the researchers and the user movement representatives. Codes and illustrating quotes were discussed in the mixed pairs, and later in the larger project group, at two occasions. The compiled analysis from the live coding was compared to the transcribed recordings by the first author. The benefits of such a strategy of combining live coding with the coding of transcripts has been discussed in previous research (33). Following the submission of key points, quotations and categories related to the analytical framework by the smaller teams, an operative group of three researchers summarized the data sent in. The analysis was then discussed at a meeting where all were present and then the summary analysis was accordingly revised with the aim of creating a consensus document which would serve as the basis of the study results.

In order to explore barriers and facilitators for including user knowledge perspectives in mental health practice, data has been approached deductively, applying a 3 fold conceptual framework based on the theory on epistemic injustice and the three aspects described above. Table 3 illustrates how these themes have been generated through the organization of data in sub-categories and categories. The results are presented below in categories which emerged in the analysis in relation to the deductive focus in the workshops on barriers and facilitators for the legitimacy of user knowledge in mental health practice. Along with the descriptions of the categories, citations from participants are specified with numbers 1–28.

**Table 3:** Themes, categories, sub-categories and examples of codes.

**Categories**	**Sub-categories**
**The theme of Testimonial (in)justice–the value**	
**and legitimacy of user knowledge**	
**Barriers and challenges** For the legitimation of user knowledge	Lack of knowledge and commitment among decision-makers Insufficient establishment with front line managers and staff
	Stigmatizing beliefs
**Factors promoting the** legitimacy of user knowledge	Representativeness Describing and raising awareness of effects
	Formalized interventions based on user knowledge
	Ongoing cultural change
**The theme of Hermeneutical (in)justice - the fit of formats and concepts**	
**Barriers and challenges** for conceptual fit of user knowledge	Illness and deficit-focused mental health service models
	Organizational instability
**Factors promoting** shifts in conceptual frameworks	Safe and stable working environments User knowledge-based methods/materials
	Integration of a variety of knowledge perspectives
**The theme of Participant based (in)justice–co-production on (un)equal terms**	
**Barriers and challenges** for equal inclusion of user-knowledge perspectives in co-production	Unequal and unjust allocation of resources Professionals own the agenda Domination of top-down approaches
**Factors promoting** partnership between equal epistemic agents	Mutuality of commitment Stable resources
	Organizational infrastructures for systematic user involvement

## Findings

A number of barriers were identified relating to the three forms of knowledge injustices, connecting to the value of users' knowledge, the integrability of such knowledge perspectives within the prevalent conceptual paradigm, and user groups' access to influence in service and system development (see Table 3). However, the current data also provides a rich set of descriptions of positive progress and of factors and strategies supporting a more epistemically *just* development. The “in” prefix in *injustice* has therefore been placed in brackets in order to indicate that the concepts are applied to elucidate barriers as well as supporting phenomena. The three forms of epistemic (in)justices are to some extent intertwined but they have provided fruitful themes for representing key findings and presenting barriers and factors promoting integration of users' knowledge perspectives in the service system.

### Testimonial (in)justice–the value and legitimacy of user knowledge

In the analysis, issues concerning the legitimacy of user knowledge are described in the theme *Testimonial injustice–the value and legitimacy of user knowledge*. This theme involves interviewees' perspectives on issues that hinder or enable user-knowledge to be validated, requested and taken into account in knowledge processes.

#### Barriers and challenges for the legitimation of user knowledge

From the discussions, it was clear how a general *lack of knowledge and commitment among decision-makers* to include user perspectives is both an effect of, as well as a contributing factor to low legitimacy of user knowledge. It was clearly noted that progress was underway, but still person-dependent and relying on individual enthusiasts:

It's often very much about who is in charge and what response you get from the leadership and whether this is taken seriously and there is commitment to drive it further. And often it is dependent on individual enthusiasts, which can also make it quite complicated (1).

Due to this general lack of recognition of the value of user knowledge, inclusion of user knowledge was rarely based on any needs analysis or perception of necessity for quality development with regard to practice. Many expressed great frustration that user knowledge was most often merely regarded as a welcomed bonus when offered without conditions or costs: “*The leadership must stand up for this becoming part of ordinary practice. We must move beyond the idea that this is something extra to regarding it as part of our core mission”* *(**9**)*. A problematic issue commonly noted as contributing to limited commitment among decision makers was the fact that user involvement initiatives were often tested in the format of projects with poorly defined expectations and that were too time limited for any positive results to become evident. The opportunity for user knowledge perspectives to be integrated and legitimized was consequently undermined. One interviewee described project funds as a “*poisoned gift*” (11) noting how there was a tendency for the project idea to be regarded as poor or without clear results when, in fact, it had not had the chance to be properly tested.

Many highlighted how laws, policy and guidelines emphasize that user knowledge perspectives need to be included in service development and delivery, but how it seems that decision-makers still lack basic knowledge on user involvement and regard it as an optional practice:

It is a bit strange that already in guidelines over ten years ago it was stated that shared decision-making should be prioritized, but then… if you asked among really knowledgeable people in some regional contexts and development leaders, no one could really explain what it meant, so no one really knew what shared decision-making was (1).

Some suggested that decision-makers believe that they already live up to these guidelines by consistently focusing on the needs of users. This lack of understanding of what user involvement implies would then explain the lack of urgency for actually including users as knowledge agents. As one interviewee noted: “*We think that we always have had a user perspective, but it's something else to work from the users' perspective”* *(**28**)*. It was commonly noted how the value of user knowledge was prioritized in system level documents but that this focus and organizational commitment was *insufficiently established with front line mangers and staff*. This was closely correlated with a lack of implementation efforts aiming to put policy into practice:

Nowadays it is well established at higher levels and also politically correct that you have to make sure that you have user influence. Also, you have well formulated policy documents…. But the problem is rather to achieve anchorage downwards in the organization, to get these policy documents and establishment on the higher levels to seep down so that it reaches the individual user.... because that's where it may really have an impact (1).

It was also evident that *stigmatizing beliefs* about people with mental health problems contributed to the low legitimacy of these knowledge perspectives. Some spoke of the historical power imbalances and the lingering notion that staff should have a monopoly on knowledge. Some described how they were sometimes appalled by attitudes among staff who could, for example, declare how users were manipulative, untrustworthy and lacking in judgement. While it was noted that many staff members appreciated listening to recovery narratives in the context of staff trainings, this interest did not transmit to increasing their confidence in the users in their own services as competent knowledge bearers.

It was commonly highlighted how service users tend not to give weight to their own knowledge perspectives and view themselves as competent carriers of knowledge. Accordingly, self-stigma constituted a problematic aspect contributing to low legitimacy of user knowledge and thereby to testimonial injustice. One interviewee noted how “*users also need to discover that they have knowledge”* (21). Another interviewee said: “*Our users also often have very low self-esteem, are not used to being listened to and taken seriously, so you also have to work with self-assertion”* (9).

#### Factors promoting legitimacy of user knowledge

From the analysis, it was evident how *representativeness contributes to legitimacy*. Interviewees from both groups underscored how user knowledge needed to be “*valid for many”* (11) in order to be considered legitimate in knowledge processes on organizational or system levels. Preferably, knowledge processes should be anchored in the user organizations so that user representatives bring a “*palette of perspectives* (11)” into collaborative practice with professionals. Many noted how systematic methods such as UFM provided a fruitful strategy for presenting perspectives that represent experiences of a collective:

UFM has raised the status of user knowledge. It feels like the user monitors' knowledge is valued higher as it is based on a group of users' experiences and not “just” their own. They have gathered what a group thinks, because otherwise, the user representatives usually get accusations like “What evidence do you have? You are only drawing from your own experiences” (7).

Many comments reflected how *describing and raising awareness of effects contributes to legitimacy of user knowledge*. The importance of advancing the research base on outcomes was identified to motivate implementation of interventions based on user knowledge. As noted by one interviewee*: “research and data that show that these are success factors in different ways, we will need that* (5)”. In addition, the importance of not only building a research base but of consistently *disseminating* research evidence on outcomes was highlighted. Professionals who have first-hand experiences of positive outcomes, sharing good examples of how user knowledge has specifically benefited their practice, was also considered important. It was noted how “decision makers need to realize the value through concrete examples” (10). As one interviewee expressed:

... to get people to understand and show how user influence at the individual level, and also at the overall level, how it streamlines care and support processes, that is, how what you do becomes much easier and of higher quality if you make use of users' knowledge (1).

Interviewees described how a one-sided rights-perspective was insufficient for user knowledge to gain legitimacy. One interviewee noted how such a perspective sometimes drew attention away from efficiency gains:

It is very common to talk about user influence as a kind of benevolent human right, which it of course is, but there is also something that is often forgotten, that it actually makes care and support more efficient (1).

It was noted that recognition of user knowledge among professionals could not be forced but how time must be allowed for managers to discover the benefits for the quality of their own practice and ultimately for the experiences of the users of their services. Not least, it was observed how implementing user knowledge perspectives has supported alliance-building with clients:

The lived experiences have contributed to a more open climate in conversations with the clients… making it easier to reach people… we have access to a unique perspective that we then simply realize we cannot be without (8).

From the interviewees' descriptions, it was evident how legitimacy and testimonial justice was strengthened through the use of *formalized interventions based on user knowledge*. The value of user knowledge was discerned when mediated and applied within the frameworks of methodized approaches (such as UFM, peer support work or materials such as The Recovery Guide). In services where such methods had been successfully implemented, user knowledge perspectives had gained legitimacy and were systematically shared in client work, in staff training and in dialogue with staff.

Many interviewees noted how members of staff who had positive experiences of user involvement through various formalized initiatives typically acknowledged user knowledge as an invaluable element of an evidence-based practice:

How cool it was that when we had a number of employees who were involved in this project and when they returned saying “how are we going to be able to work in any other way than this?” It was so incredibly natural that this evidence-based social service or knowledge-based social service, [were to include] that third component. It became so natural in all activities (6).

From the descriptions of the interviewees, it was evident how the user knowledge perspective was indeed steadily gaining legitimacy by an *ongoing cultural change*. While it was generally acknowledged that there is much work to be done for user knowledge to be fully legitimized, many noted a slow but positive development occurring over time, and expressed how a long-term view was necessary in order not to be discouraged by slow results. One interviewee noted, for example, how initiatives cannot be regarded separately, but that a variety of simultaneously occurring elements are “*pulling in the same direction*,” such as “*educations, policy development, research and an increasing focus on person centered care”* (13). While, as previously noted, problematic aspects of the short term projects was commonly discussed, some interviewees reflected that they might also be a contributing factor to this progress. Concordantly, it was noted as a fruitful approach for the user movement to direct resources toward services who were genuinely interested: “*where doors were already open or half open”* (3), who had autonomously begun promoting user influence. This approach may be understood as a way of tapping into the energy and this current of cultural progress.

### Hermeneutical (in)justice-the fit of formats and concepts

Many descriptions in the data reflect problematic aspects of integrating users' knowledge perspectives within the formats for knowledge predominant within welfare and healthcare systems. In the analysis, issues relating to this lack of conceptual fit with the prevailing paradigm have been sorted into categories and collected within the theme *Hermeneutical (in)justice-the fit of formats and concepts*. Interpretive frameworks operate in a given context, that steer and delimit how we organize, order and navigate the world. It was clear from the analysis that different knowledge perspectives honored divergent understandings and values in relation to mental health and recovery.

#### Barriers and challenges for conceptual fit of user knowledge

From the discussions, it was discernable how *illness and deficit-focused mental health service models impede the desired paradigm shift*. Many comments reflected a poor fit between user knowledge perspectives, often expressed as narratives based on holistic views on health, illness and recovery that do not fit in with the welfare organizations that are structured based on diagnostic classifications and quality standards that relate to symptom relief, care consumption and compliance. One interviewee noted that the prevailing “*interpretive prerogative”* (7) granted to professionals constituted a particular challenge for such a shift. Many highlighted the stereotypical staff and user roles as a problem. As an illustrative example of such an “us and them” mentality, one interviewee described how staff at services about to implement peer support could ask “*where will the peer supporter sit and have coffee?”* (3). Accordingly, the conceptual spotlight of hermeneutical (in)justice drew attention to a discourse perspective, shedding light not only on how specialist knowledge outlines the boundaries for spoken and written language, but how it also generates and maintains structures, organizational logics and indicators of quality. From the interviewees' descriptions, it was evident that professionals were often unaware of these barriers, that they did not appreciate the importance of the user movement developing and implementing their interventions independently and delivering them in the formats that harmonize with the value base and knowledge contributions of users. One interviewee describes risks of user knowledge being co-opted and colonized by the prevalent paradigm based on deficit-based perspective on mental health:

They want to cherry-pick-take over the methods developed by the user movement and run them themselves. With no understanding of the value of independence. Push it into diagnosis-based manuals. We have to reconquer recovery by means of The Recovery Guide (4).

In analyzing the data, it was also notable how interviewees rarely spoke about the influence of user representatives in terms of them being knowledge bearers. Whilst the questions were directed at barriers and facilitating factors for implementing user knowledge perspectives, responses commonly shifted focus toward technical and structural issues of implementation. This failing to construe the contributions of users' knowledge perspectives, even amongst those most committed to user involvement, may reflect a general lack of conceptualization of user knowledge within the interpretive frameworks prevalent in the welfare system.

It was commonly noted how change that requires quite radical restructuring of mindsets, as well as of practice, was hampered by *organizational instability*. As one interviewee noted, “*high staff turnover requires that attitudinal issues are constantly processed, and that staff training is continually repeated”* (12). In addition, it was noted by many how reevaluation of prevalent ways of thinking and working requires an openness to criticism. One interviewee reflected that paying heed to critical perspectives seemed easier for external, top-level decision-makers than for managers and staff actually performing the practice that is often subject for criticism: “*The closer you are to the services and the users, I can experience that it is more difficult to accept criticism”* (6).

#### Factors promoting shifts in conceptual frameworks

As noted, organizational instability was expressed as a barrier for introducing new perspectives. In concordance, a *safe and stable working environment*, where staff felt secure in their working roles was highlighted as a supportive factor. Interviewees' comments on this issue may be understood to reflect how a change of practice, that requires accommodation of new paradigms, takes time and space for people to reflect and process:

You need to process it a bit before… as a staff member you are in the middle of something and you think that what you do is probably right and proper and so on, you need to process it about a bit in the workplace (1).

It also requires courage, especially when those novel perspectives may be challenging prevalent beliefs amongst staff that they have been performing their work according to best practice: “*If you have a staff group that feels good at work, I also think that it is easier to dare to let in other methods or dare to see things in new ways”* *(**26**)*.

Many interviewees reported successful implementation of manualized interventions based on user knowledge. Common to these interventions was that they provided the holistic, recovery-oriented, bottom-up perspectives of service users with *knowledge-based methods/materials geared to prevailing structures of the mental health system*. Amongst these examples, the Recovery guide was highlighted. Since it is based on a recovery perspective, providing a holistic perspective on mental health and recovery, it postulates a bridging over organizational barriers:

The Recovery Guide, of course, where we work more in a recovery-oriented way, where our employees gain knowledge about recovery, that we not only “store” patients and medicate patients, but it is about so much more and where patients then become very involved in their care and support, which of course they should be, it's their recovery process (3).

Likewise, UFM was highlighted as a formalized method structured according to the prevalent organizational logic that similarly to the recovery guide “*demanded co-operation”* (13) across organizational boundaries. It was also noted how these user-led mental health service evaluation processes commonly brought about constructive dialogues for improvement between user movement- and service representatives. Peer support workers, who according to the Swedish, user movement driven model, bring a broad and collective user knowledge base to their practice, further generated quite radical shifts in perspectives in the staff groups. One interviewee described how staff had become aware of and raised alarms about problems in service provision, noting how “*there had been some stormy awakenings”* (10).

From the discussions, it was evident that ongoing shifts in culture were not driven by adding experiential knowledge to professional expertise but through *the integration of a variety of knowledge perspectives*, that had the potential to synergistically expand spheres of knowledge. For example, some interviewees spoke in terms of fruitful co-learning processes that occurred when staff and users attended recovery trainings together:

… Going together with staff is the best! We have seen that this co-learning has had an effect. Before, we had all our training in a recovery-oriented approach for staff, but then we realized that if we include people with their own experience and they go together, it is far more rewarding (4).

Overall, many comments related to the positive effects of integrating holistic perspectives in organizational structures that served to promote a shift toward a holistic and recovery-oriented paradigm.

### Participant based (in)justice-co-production on (un)equal terms

Comments that relate to issues of influence and power distribution in knowledge processes, reflecting whether or not user representatives participate in equal partnerships are sorted in categories and sub-categories together making up the theme *Participant based (in)justice-co-production on (un)equal terms*.

#### Barriers and challenges for equal inclusion of user-knowledge perspectives

Interviewees consistently highlighted a variety of power asymmetries that hindered user representatives from participating as equal partners in knowledge processes. A lack of resources in terms of time, money, people and administration was commonly highlighted as a major barrier for user involvement. It was evident how user movement representatives were constantly in a position of disadvantage in the face of the *unequal and unjust allocation of resources*. Even though it was commonly acknowledged that resources were limited at all organizational levels within the mental health system, influence over resource allocation resided, to a greater extent, within the realms of professionals than with the user organizations for which resource scarcity was noted to be particularly challenging:

There has also been uncertainty about financing. It is always difficult when you try to run a larger operation that costs some money. Now we have some incentive funding, but they are often for one year at a time, it is not very stable to build on (8).

It was commonly noted how the user movement had low priority and was often subject to budget cuts. It was also noted that professionals received their pay when collaborating with user movement representatives during workdays, while the latter worked for free, causing strains on the user organizations and limiting the possibility to harness the potential of user knowledge: “*There is so much we could do to make use of and build on this knowledge, but we do not have the resources to manage”* (21). Many aspects of unequal allocation of resources were discussed, involving user movement representatives having less insight into the system, overview of the services and the decision-making routes, less access to established roles and functions in the system. Particularly, many noted the challenge of finding and preparing individuals that had the desire and capacity to participate.

As a major barrier for equal-terms partnerships, interviewees highlighted the lack of awareness amongst professionals of the disadvantaged position of the user movement in relation to power, resources and decision-making:

They want to ride the train, but they do not want to pay for laying rails. They don't understand that the user movement needs some kind of infrastructure to be able to exist and run their services, they think we only consist of people who have as a hobby to come to a meeting a little now and then (29).

It was noted by another interviewee how “*professionals in possession of power did not perceive that they have the power, but they do, since they have the legislation in their hands”* *(**7**)*.

The position of disadvantage of user representatives was also commonly highlighted in relation to the ways in which *professionals set the agenda* and delimit user movement autonomy. It was, for example, observed that professionals could specify which user representatives were invited to collaborate and under which terms collaboration was to take place. While acknowledging the importance of involving the “*right persons”* (19) in shared deliberation, interviewees observed an unwillingness of some professionals of even associating with the user movement:

We notice that they want to pick out individuals from the user movement who they think are at the right level, so they do not want to associate with the user movement… but they prefer to pick-and-chose people with whom to communicate (19).

This proneness amongst seemingly committed professionals to fail in actually inviting users in knowledge processes that concern them was commonly noted. The following quote reflects the inclination to act as interpreters of users' values and needs, rather than inviting them to the table: “*But what creates value for the clients in these different contexts? And where are the ones we should ask what was value-creating? They are not invited”* (6). Similarly, it was observed how the user movement did not have power over the agenda and that professionals sometimes “*wanted to steer discussions”* (17) or delimit the issues in which users could have influence to insignificant matters. The occurrence of such tokenistic practice is exemplified in the following quote:

So it was clear that the user organizations wanted to have influence in issues important to them, such as appointments of staff, while psychiatry thought yes, but it works so well if they can have a question-box and decide the color of the curtains, for example, which was much easier to take on board. So, I think that the willingness to let go of power and control is an important issue (7).

Another phenomenon noted that might be understood in terms of tokenism, was staff applying methods designed to support user influence in such a shallow way that no genuine sharing of power took place and user influence thus remained superficial. It was commonly discussed how the position of dependency of user representatives implied a need to adapt an agreeable and non-confrontational attitude in order to be invited to collaborate and thereby implicating a risk for co-optation. Handling this predicament was commonly described in terms of a tricky balancing act of being a “*critical friend”* to psychiatry (30) or “*To not get coopted but at the same time not be too confrontative”* (6). Expressing critical perspectives was often done at the risk of being excluded from collaboration:

It can be a difficult balance between being a representative of a user association and at the same time being compliant with psychiatry. When, for example, user representatives have written debate articles that have a strong negative view of psychiatry, they may be deliberately excluded from working groups, influence councils, etc. (6).

In the face of this dilemma, many observed the need for some user movement actors to maintain an independent stance and for others to be more consensus-oriented in order to enable collaboration. Others described their endeavors to gain influence in knowledge processes from an unfavorable position in terms of having to persevere in the face of resistance from professionals. The importance of persistence and patience was noted in order to: “*horn oneself into various contexts”* and “*press in the practice of involving user perspectives”* (1).

Despite the intentions of those dedicated to respect user perspectives, many interviewees noted the risk of maintaining a *domination of top-down approaches*. Many appreciated how a greater power balance had indeed been achieved through NSPH as a national, well-resourced user movement organization. However, amongst the NSPH representatives participating in the FGIs, some noted the risk of NSPH becoming too established and thereby “*gaining a monopoly”* (29) on influence work and losing the rootedness amongst local user representatives.

In discussing power dynamics in relation to top-down approaches, it was underscored how staff too need to feel empowered in order to realize partnership and fair play. As one interviewee noted: “*Influence is also needed for the staff, so that they also feel that they have influence in these development- and change processes, as well as the users”* (2).

#### Factors promoting partnership between equal epistemic agents

From the discussions it was evident how *mutuality of commitment* was a prerequisite for equal partnership. For example, equality was supported in cases where user movement representatives could be involved in setting the conditions for collaboration. Accordingly, the analysis brought to light how accountability mechanisms sometimes were at work, supporting partnership and participation on equal terms. This occurred when formalized approaches for implementing user knowledge included some sort of mandatory counter performance. For example, as the following quote implies, access to the recovery guide material requires counter-performance from services: “*Now that we get requests from other regions, we have a whole list of things they need to commit to if they are to implement the Recovery Guide”* *(**30**)*. Likewise, it was observed how UFM processes were more likely to lead to user influenced development work when an obligatory follow-up assessment was included in the commission:

As enabling factors I would say … follow-ups of the UFM, and reviewing how has it affected the services, if they have made any changes, etc. Getting such questions makes them adhere to the recommendations (29).

In addition, it was noted how inclusive efforts needed to be employed by mental health system actors, going beyond inviting user representatives to join in *their* initiatives on *their* home turfs, but instead reaching out to people in their organizations and forums. As one interviewee noted: “*If they [the young service users] won't come to us, we have to find them and come to them.”* (21).

Throughout the discussions, it was stated that equal partnerships required *stable resources*. The mandate to implement user influence requires that finances be budgeted for the work it involves in achieving systematic and structured partnerships. From the discussions, it was evident that such access to resources varied greatly between regions in Sweden. User organizations located in regions in which they were provided a steady inflow of resources had the possibility to establish sustainable structures, following concrete action plans and working proactively with the implementation of distinct methods:

The success lies in the fact that it is a concrete way of working. So, it becomes a clear structure in how we should work with project groups and with steering groups and that there is a mandate to drive things forward. Before it was not so clear and then it mostly felt like we floated around (4).

As noted earlier, it was evident from the discussions how an ongoing cultural change is underway and how *organizational infrastructures for systematic user involvement* sometimes were in place, e.g., with user “*involvement embedded in management systems, ensuring sustainability”* (11). Earlier, the purpose of involving user perspectives had often been vague, but there is now a strategic thinking on what goals are to be achieved, on which actors should participate and what target groups needed to be reached:

It didn't have any real purpose before [when user representatives participate in seminars]. Only information stacked on top of each other. Now there is strategic thinking about who should participate and listen. Now it feels like we're talking purpose (20).

Interviewees reflected on this progress noting that user representatives to a larger extent were now involved in entire development processes, from planning to follow-up stages. They were also more often involved in choosing which issues needed to be addressed and in which arenas collaboration was to take place.

Ideally, it was observed, that the “*user movement itself was strong enough to carry”* (5) their work. Otherwise, it was necessary that they were provided financial support but also other opportunities for education and team- and leadership training: “*We make sure that they get paid for travel and that they receive training. So that you do not come in with a knowledge deficit”* (10).

One success factor, related to organizational infrastructure was suggested to be the employment of user representatives with decisional mandates at system levels within the service organizations. As one interviewee noted, such a role implied having access to decision-makers and infrastructure and being able to independently move processes forward without dealing with gatekeepers:

The biggest success factor is getting a user in at the system level. You have access to all decision-makers. You can run the work independently and do not have to toss around so much, just to get an approval (31).

Another interviewee noted how being co-located in the same corridors as staff and managers created a breeding ground for co-learning and co-production. In cases where such organizational infrastructures were in place, it was noted how an improvement in quality was evident, regarding the care as well as the working environment: As one interviewee concluded:

Better care and better working environment. We can see this in evaluations. It produces a different climate in the discussions in the working groups, it breeds a better working environment (11).

## Discussion

**Figure 1:**
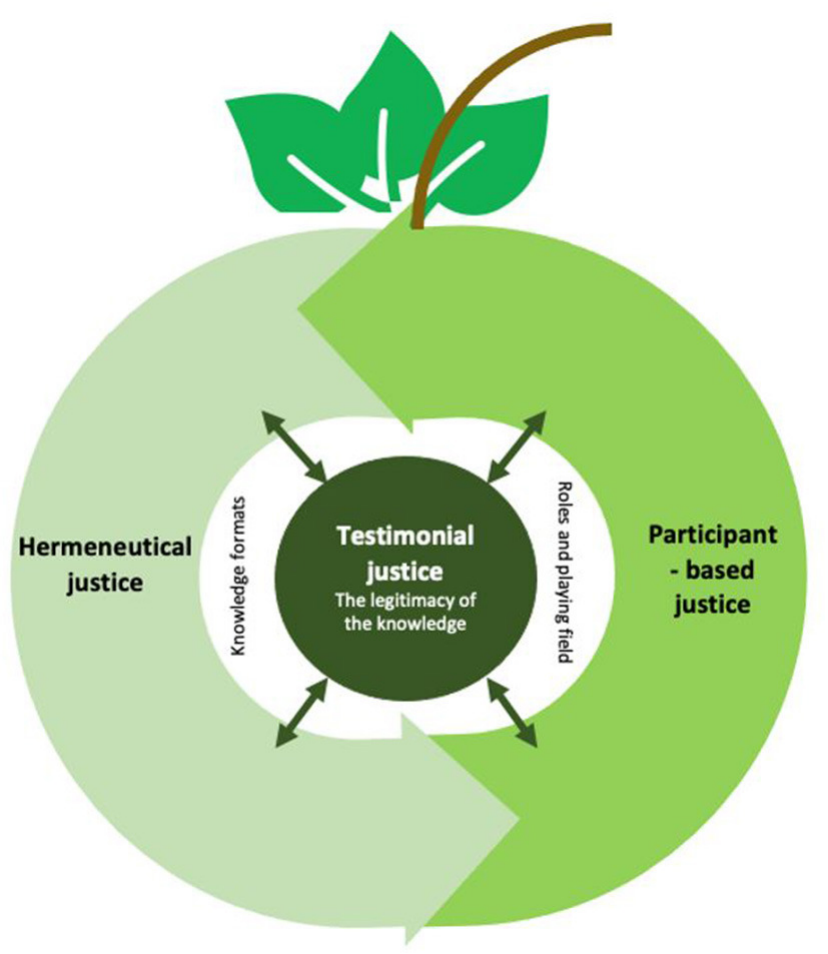
Model of interrelationships between the different forms of knowledge (in)justice.

The analysis has clarified central barriers and facilitators to the legitimacy of user knowledge in mental health practice, applying the theoretical framework of epistemic injustice (6, 20, 22). In order to illustrate the three aspects that structure the analysis we suggest the following model (Figure 1) which has taken the form of an apple, as a metaphor for our focus on knowledge. It attempts to describe the interrelationships between the different forms of knowledge (in)justice. While a simplification of what is clearly a complex process, with many contributing factors that are not included here (meta-level issues regarding economy and resources for example), we found the model useful in both reflecting the interactivity in these concepts, and as a structure for considering these various aspects in the practical application of the results for future projects.

At the center of the model (the apple's core), we have placed *testimonial justice* which refers to the extent to which user knowledge is seen as legitimate, valued and credible at an individual and collective level. The extent to which a higher level of testimonial justice is achieved relates to the other justice forms, and is both influencing and being influenced by these. *Participant-based justice* relates to a more equal playing field, where different actors play on equal terms, with equal team structure and equal power distribution, in a joint construction of knowledge. Participant-based justice also has a two-way connection to *hermeneutical justice*. The knowledge formats and terminologies that are dominant and how quality and competence are defined in mental health systems is a critical focus for the constitution of an equal playing field for knowledge formulation, but also to the legitimacy of user knowledge. The double-sided relationships between all three forms of knowledge justice means that increased equality in one of these, have significance for all forms of knowledge justice. Altogether, the three knowledge (in)justices describe both barriers and facilitators that affect the extent to which user knowledge is legitimized and integrated in mental health practice.

### A movement toward knowledge legitimacy and integration

The research team, as well as the interviewees, represented diverse perspectives, and many were able to place the discussions within a broad, historical context. A historic movement toward increased user influence was discussed in the workshops: From being seen as “disturbing” and critically opinionated patients, to being invited in to share stories of illness and recovery, to the position of the user as a “competent customer” and slowly progressing toward being seen as valuable partners in developing relevant services. In accordance with previous literature, the discussions centered on the idea that we are now in the midst of a progression from “influence” as the vision–to one in which a position as partners in coproduction has begun to dominate the discourse for practice, research and development of services (8, 34).

Co-production and co-learning were dominant in discussions of both successful examples and of factors important for the future. These types of knowledge-based contacts, where users and professionals received and produced knowledge in a partnership, seemed to directly impact the legitimacy of user knowledge. As the model above suggests, this process is complex and multi-directional, but inviting professionals and users into a learning context where diverse knowledge perspectives can meet, also contributes to increasing respect for user knowledge and a future willingness to integrate this knowledge form. The benefits of such joint knowledge production activities remain to be explored (35) but the current discussions highlighted the value of relation-building, and of how proximity or co-localization provided breeding grounds for dialogue and a spurring of change processes.

Closely aligned with this shift in vision for how patients or users might co-produce rather than just influence services, was a focus on how issues of influence might be viewed from a citizen, rather than user perspective, in a democracy context. From this standpoint, the issue of representativity was discussed in relation to the role of user organizations and the rights of individuals who may not choose to or feel represented by these organizations. The findings highlight previously noted risks of primarily involving participant ready individuals in that it may limit diversity (18, 36). Representation being a crucial aspect of democratic practices (37), the findings indicate that increased efforts are needed to ensure broad representation in order to uphold the democratic aim.

### Systematic methods and relevant outcomes

The results suggest that the systematic methods which were an impetus for the research project, were considered by the interviewees and the research team as particularly effective for increasing the legitimacy of user knowledge and implementing user influence over time. Building methods for integrating and disseminating user knowledge (The Recovery Guide, UFM), as well as for providing services (Peer Support) were described as turning points for services who had previously committed primarily ideologically to user involvement and that could now integrate a concrete component in their practice. Although we cannot confirm the success of particular systematic methods in furthering the legitimacy of user knowledge, the framework suggests that developing specific forms of delivering user knowledge within the mental health system can function as a critical aspect in implementation processes. It was noted that the structural fit of these interventions provided a central facilitating factor. Working together with researchers to demonstrate outcomes of these methods for users and system quality improvements is recommended. This could potentially contribute to demonstrating the “added value” that might be associated with increased attention to user knowledge, which may support making user involvement a priority in economically stressed organizations. Simultaneously, awareness should be raised of the risks of professionalization and cooptation that are associated with such methods that involve close relationships with authorities. This might imply a neutralization of charged issues of importance for many service users, not least individuals struggling in the margins of society (38).

### Financing and sustainable structures create legitimacy

The results suggest that the sustainability of initiatives to increase the legitimacy of user knowledge in mental health services is connected to the organizational and financial possibilities for doing so over time. Economic compensation for users who are not employed by the system is essential. The lack of compensation for the user representatives who contribute to advisory committees, work on quality assurance and development projects for example, makes them even more vulnerable to knowledge injustice. Permanent, rather than project-based funding was also considered crucial for stable implementation. Agreements with government agencies, national authorities and local and regional actors provided support for these user-based knowledge methods. In the end, as with many other change processes, the financing and responsibility for these services must come from the highest levels.

The results further suggest a need for stable welfare organizations and secure working conditions for staff, as a prerequisite for change. Readiness for change is also connected to information, communication and the involvement of front-line staff, as well as the leadership, in these change processes. The discussions also pointed to the fact that working environments not only relate to implementation questions. Many program representatives also described positive effects in the working environment of staff when a more user-inclusive culture developed.

### Legitimizing knowledge is a process and power issues predominate

There is no one, static answer to the question of developing legitimacy for user knowledge. Knowledge is created over time, in contexts that influence the process. The opportunities for real participation in these contexts require shifts in power structures so that new forms of knowledge and new collaborations for learning can be integrated in mental health practice.

An important issue reflected in our results was the precarious balancing act user representatives had to perform in order to participate. They had to negotiate the tightrope of being cooperative but not too compliant and in providing fresh perspectives without being too critical. These findings resonate with previous studies focusing on user involvement of individuals in their care and support (6, 39). It further highlights the need to develop conditions and methodologies for an open exchange of experiences and opinions in order not to silence voices and miss out on important knowledge perspectives.

The results also suggest that perspectives on integrating user knowledge and allowing for influence are affected by attitudes that may not be readily apparent when implementing initiatives building on user knowledge. Interviewees described a “we already do that” mentality where staff perceptions of having succeeded in focusing on user influence were not necessarily reflected in users' experiences of having their perspective legitimized and included. Users themselves often lack confidence in their role as knowledge-bearers, and therefore maintain a passive voice, even when services are initially seeking their voice. Self-stigma may be thereby constitute an obstacle to participation. Even at the individual level therefore, epistemic (in)justice is worth considering when developing user influence.

## Conclusions

The model presented above can be seen as an explanatory framework for understanding the complexity of legitimizing this unique form of knowledge in mental health services and thereby supporting user influence. It may also be seen as a framework for action, as it has emerged from the discussions we have had on an ongoing basis in the co-production team. The study and analysis have clarified, using the theoretical framework provided by epistemic injustice, many of the strategies that the user movement representatives have successfully struggled to develop in their work. The systematizing of a collective user knowledge base, presented in a form (method), that is relevant and adapted to a psychiatric context has characterized the specific methods we have considered. The issues of participation and power have additionally served to confirm the need for access to the “playing field” in a sustainable fashion, if the development and “packaging” of the knowledge is to stimulate a process that continues beyond the initial presentation of this knowledge. This was very clearly exemplified by the “Recovery Guide” implementation in which the authors in the user movement negotiated access to the mental health service in order to manage, follow-up and evaluate the implementation process.

The current analysis points to the legitimacy of user knowledge as related to the issue of representativeness, the systematic inclusion of this knowledge in applicable methods, stable resources, positions within the mental health system and participation in the process of integrating this knowledge base in mental health contexts. The results suggest that the focus must shift from the current paradigm, which primarily involves the importing of this knowledge into a professional system, to one in which the mental health system, including the national authorities, actively participate in developing cultures and organizational structures in which this knowledge base is valued and integrated in mental health practice. Further research is needed into how more isolated and independent users may gain influence and have their positions as active knowledge-bearers confirmed in practice.

## Data availability statement

The raw data supporting the conclusions of this article will be made available by the authors, without undue reservation.

## Author contributions

DR managed the research project and had main responsibility in designing the study. KG had the main responsibility in completing the analysis and writing up the manuscript, working in close collaboration with DR and HN. In these later stages, all authors took part in the analysis process in connection with group meetings and in reviewing and revising the manuscript. All authors were involved in developing the interview-guide, in performing the data collection and in the first stages of analyzing data.

## Funding

This study was funded by the Swedish Research Council for Health, Working Life and Welfare under [Grant Number: 2020-01297].

## Conflict of interest

The authors declare that the research was conducted in the absence of any commercial or financial relationships that could be construed as a potential conflict of interest.

## Publisher's note

All claims expressed in this article are solely those of the authors and do not necessarily represent those of their affiliated organizations, or those of the publisher, the editors and the reviewers. Any product that may be evaluated in this article, or claim that may be made by its manufacturer, is not guaranteed or endorsed by the publisher.
